# A Real-World Pharmacovigilance Analysis of the Safety Profiles Associated with Anti-MRSA Agents Using the Japanese Adverse Drug Event Report (JADER) Database

**DOI:** 10.3390/idr18030043

**Published:** 2026-05-02

**Authors:** Yuki Hanai, Shusuke Uekusa, Mizuki Mori, Kohei Shimoyama, Hayato Ohashi, Koji Nishimura, Sachiko Yanagino, Takahiro Matsumoto, Kazuhiro Matsuo

**Affiliations:** 1 Laboratory of Clinical Infectious Diseases and Therapeutics, Meiji Pharmaceutical University, Tokyo 204-8588, Japan; y-hanai@my-pharm.ac.jp; 2 Department of Clinical Pharmacy, Faculty of Pharmaceutical Sciences, Toho University, Chiba 274-8510, Japanmatsuok@phar.toho-u.ac.jp (K.M.); 3 Department of Pharmacy, Toho University Omori Medical Center, Tokyo 143-8541, Japan

**Keywords:** anti-MRSA agent, pharmacovigilance, adverse event, Japanese Adverse Drug Event Report (JADER) database, observational study, retrospective analysis, Standardized MedDRA Query, Japan

## Abstract

Background: Anti-MRSA agents are essential for treating severe infections, yet their use is constrained by distinct toxicity profiles. However, comparative real-world data remain scarce. Methods: This nationwide pharmacovigilance study used the Japanese Adverse Drug Event Report (JADER) database (2004–2025). Disproportionality analyses (proportional reporting ratio [PRR]) were performed at the Standardized MedDRA Query and Preferred Term levels, complemented by Weibull-based time-to-onset modeling, to characterize AE patterns associated with vancomycin (VCM), teicoplanin (TEIC), arbekacin (ABK), daptomycin (DAP), linezolid (LZD), and tedizolid (TZD). Results: Distinct agent-specific AE profiles were observed. VCM showed disproportionate reporting of acute renal failure (PRR 6.66) and severe cutaneous reactions. TEIC displayed fewer renal signals but relatively higher reporting of hematologic events (PRR 3.51). ABK demonstrated high disproportionality in acute and chronic renal failure, reflecting aminoglycoside nephrotoxicity. DAP showed a high reporting signal for eosinophilic pneumonia (PRR 23.30), interstitial lung disease, and creatine kinase elevation/rhabdomyolysis, with wear-out hazard patterns suggesting a possible time-dependent reporting tendency. LZD exhibited hematopoietic signals (PRR 6.13) and additional associations with hyponatremia, lactic acidosis, and optic neuropathy, consistent with marrow suppression and mitochondrial toxicity. Weibull analysis indicated cumulative “wear-out” risks for renal, hepatic, and hematologic events, whereas hypersensitivity and many pulmonary events followed random-failure patterns. Conclusions: This large-scale JADER analysis delineated the distinct safety profiles of the six anti-MRSA agents. The key findings included DAP pulmonary and muscle toxicities, LZD hematological events, and VCM nephrotoxicity. Time-to-onset modeling indicates potential cumulative versus random risk patterns, suggesting the need for individualized monitoring and cross-validation.

## 1. Introduction

Methicillin-resistant *Staphylococcus aureus* (MRSA) remains one of the most formidable pathogens in healthcare settings, particularly in patients with nosocomial pneumonia, bloodstream infections, and skin and soft tissue infections [1,2]. Currently, several anti-MRSA agents are used in clinical practice, including glycopeptides (vancomycin [VCM] and teicoplanin [TEIC]), aminoglycosides (arbekacin [ABK]), lipopeptides (daptomycin [DAP]), and oxazolidinones (linezolid [LZD] and tedizolid [TZD]). Although these agents are indispensable for managing MRSA infections, their clinical utility is often constrained by the risk of adverse events (AEs), which vary in nature and severity among the different drugs.

Each agent exhibited a well-known toxicity profile. VCM is closely associated with nephrotoxicity, particularly at high serum trough levels or during prolonged use [3,4], whereas TEIC, another glycopeptide, tends to cause renal toxicity less frequently than VCM [5]. Similar to other drugs, ABK, an aminoglycoside primarily used in Japan, carries a high risk of both nephrotoxicity and irreversible ototoxicity, similar to other drugs in its class [6,7]. DAP has been linked to musculoskeletal toxicity, including myopathy and, in rare cases, rhabdomyolysis, thus requiring regular monitoring of creatine kinase levels [8]. LZD and TZD are known to cause hematological toxicities such as thrombocytopenia and anemia, especially during extended therapy [9,10]. Although these adverse effects have been described individually, comparative evaluations of all anti-MRSA agents using uniform patient datasets and standardized analytical approaches remain limited. Most existing studies rely on case reports, single-center experiences, or trials restricted to a single drug, leaving a paucity of real-world head-to-head data on the frequency, spectrum, and time-to-onset of AEs.

Spontaneous reporting systems (SRSs) such as the Japanese Adverse Drug Event Report (JADER) database offer valuable resources for pharmacovigilance research. Maintained by the Pharmaceuticals and Medical Devices Agency (PMDA) of Japan, the JADER database has been widely employed to investigate drug-related safety signals, including torsade de pointes, hepatic injury, and hematological disorders [11,12,13]. Despite its extensive utility, to the best of our knowledge, no study has systematically compared the AE profiles of VCM, TEIC, ABK, DAP, LZD, and TZD using this database. Elucidating the differential safety profiles of these agents is critical for optimizing therapeutic decision-making and enhancing AE monitoring strategies in patients undergoing treatment for MRSA infections.

Accordingly, this study aimed to conduct a disproportionality analysis of six widely used anti-MRSA agents using data from the JADER database and to investigate an overall safety profile. This study examined both the overall and drug-specific frequencies of the reported AEs, described their organ system-specific profiles, and assessed the time-to-onset patterns using Weibull distribution modeling. These findings are intended to provide real-world evidence to support the safer and more personalized use of anti-MRSA agents in clinical practice.

## 2. Subjects and Methods

### 2.1. Data Source and Study Design

This retrospective disproportionality analysis used data from the JADER database, covering reports submitted between April 2004 and February 2025. This research adheres to the READUS-PV guidelines to ensure transparent and comprehensive reporting of disproportionality analyses [14]. The JADER database, maintained by the PMDA and publicly available through its website (https://www.pmda.go.jp/, accessed on 16 March 2025), comprises four relational datasets: DEMO (patient demographic information including age, sex, weight), DRUG (drug exposure data), REAC (AE information), and HIST (primary diseases and comorbidities). Each report may have described multiple drugs or AEs. In the present study, only cases in which an anti-MRSA agent was explicitly designated as a “suspected drug” were included in the primary analysis; reports in which the drug was classified solely as a concomitant or interacting drug were excluded. Duplicate reports, where identified, were also excluded from the analysis.

### 2.2. Drugs of Interest

Six anti-MRSA agents were analyzed: VCM, TEIC, ABK, DAP, LZD, and TZD. Both intravenous and oral formulations were considered applicable, although certain agents such as ABK and DAP were administered exclusively via the parenteral route. This study evaluated VCM, TEIC, ABK, and DAP only when administered intravenously, whereas LZD and TZD were analyzed after both intravenous and oral administration. This categorization reflects the approved clinical practice in Japan and ensures consistency across reports. Furthermore, we included the active ingredient/brand name/salt form in the target drug group.

### 2.3. Adverse Event Definition and Classification

AEs were coded using the Preferred Terms (PTs) of the Medical Dictionary for Regulatory Activities, version 28.0 J (MedDRA^®^/J; https://www.jmo.pmrj.jp/, accessed on 13 April 2025). Classification was based on clinically relevant toxicity domains known to be associated with anti-MRSA agents, including renal toxicity (e.g., acute kidney injury, renal failure, elevated serum creatinine), hematological toxicity (e.g., thrombocytopenia, anemia, and pancytopenia), musculoskeletal toxicity (e.g., myopathy, elevated creatine kinase, and rhabdomyolysis), neurological toxicity (e.g., peripheral neuropathy, ototoxicity, and dizziness), and hepatic toxicity (e.g., liver enzyme elevations and hepatic dysfunction). Each AE was counted once per report; when multiple AEs were recorded in a single report, all relevant events were included. To complement this domain-based classification, Standardized MedDRA^®^ Queries (SMQs) (v28.0, https://www.jmo.pmrj.jp/, accessed on 13 April 2025) were employed, which aggregate PTs into clinically meaningful clusters. Overall, 110 level 1 SMQs were analyzed using narrow-scope searches to enhance specificity. This combined PT- and SMQ-based strategy ensures both the comprehensiveness and clinical relevance of AE detection. All reports were retrospectively recoded using this version to ensure consistency in event classification over time.

### 2.4. Signal Detection: Proportional Reporting Ratio (PRR)

Disproportionality analysis was conducted using a case/non-case approach. A “case” was defined as a report in which the drug of interest and an AE of interest co-occurred, while “non-cases” comprised all other reports in the database. PRRs with corresponding chi-squared value were calculated for each drug–AE pair. A signal was considered present when four conditions were met: (1) PRR ≥ 2, (2) chi-squared value ≥ 4, and (3) at least three cases, in accordance with established pharmacovigilance standards [15,16], and (4) statistical significance after multiple testing correction, defined as a Benjamini–Hochberg adjusted *p*-value < 0.05. To address numerical underflow arising from extremely small *p*-values, chi-squared test *p*-values were handled on the logarithmic scale. Multiple testing correction was then performed using the Benjamini–Hochberg procedure applied to log-transformed *p*-values to control the false discovery rate. Results are reported as log-transformed FDR-adjusted *p*-values (log FDR-adjusted *p*-values). The PRR is defined as the ratio of the proportion of a specific adverse event reported for a drug of interest to the proportion of the same adverse event reported for a comparison group of drugs. As a sensitivity analysis, disproportionality was additionally assessed using the Reporting Odds Ratio (ROR) with 95% confidence intervals and the Information Component (IC) with its lower 95% credibility bound (IC_025_) [16]. A signal was considered present when the lower bound of the 95% confidence interval for the ROR exceeded 1.0 and when IC_025_ was greater than 0. These analyses were conducted at the same aggregation level as the primary PRR analysis. Volcano plots were generated for visualization, with the x-axis representing log_2_-transformed PRR values and the y-axis representing chi-squared statistics. The size of each plotted point was proportional to the reporting ratio, defined as the number of reports for a specific adverse event divided by the total number of reports for each anti-MRSA agent, representing the within-drug proportion of each event. Points located in the upper-right region of the plot indicate signals with higher disproportionality and greater statistical strength.

### 2.5. Time-to-Onset and Weibull Distribution Analysis

Time-to-onset was defined as the number of days from the start of drug administration (recorded in the DRUG dataset) to the date of AE onset (recorded in the REAC dataset). To minimize misclassification, only reports with clearly recorded start and end dates of drug administration were considered eligible. When the onset of an AE occurred after treatment discontinuation, the event was included only if it occurred within 14 days of drug cessation. Reports with missing or inconsistent date information were excluded. The onset distribution of the AE was visualized using box plots. Weibull distribution modeling was performed to evaluate how the risk of AEs changed over time. The shape parameter (β), along with 95% confidence intervals (CIs), was used to characterize the hazard function, with β < 1.0 indicating an early failure type in which the risk is highest shortly after initiation, β ≈ 1.0 suggesting a random failure type with a constant risk over time, and β > 1.0 indicating a wear-out failure type in which the risk increases as treatment duration progresses. The scale parameter (α), reflecting the dispersion of the distribution, was also calculated. A minimum of 20 cases was required for Weibull analysis to ensure stable estimation of model parameters, in line with methodological considerations for small-sample reliability in parametric survival modeling. The goodness-of-fit of the Weibull distribution was assessed using the Anderson–Darling (AD) test.

### 2.6. Statistical Software

All data processing, signal detection, and time-to-onset analyses were performed using R statistical software, (v4.5.1, R Foundation for Statistical Computing, Vienna, Austria). PRR and chi-squared statistics were calculated using base R functions. Weibull distribution fitting was performed using the “fitdistrplus”(v1.2.6) and “goftest” package (v1.2.3). Figures were generated using the “ggplot2” package (v4.0.3).

## 3. Results

### 3.1. Basic Demographics of Anti-MRSA Agent-Related AEs

Table 1 summarizes the demographics of AE reports associated with anti-MRSA agents in the JADER database. The number of eligible reports was 2318 for VCM, 838 for TEIC, 227 for ABK, 552 for DAP, 1750 for LZD, and 39 for TZD. Males accounted for over 60% of the cases across all agents, and patients aged ≥ 60 years represented the majority, particularly those with ABK (>80%). Body weight was reported less consistently; when available, 40–69 kg was the most common category. Overall, the demographic patterns were comparable among the agents.

### 3.2. Signal Detection at the SMQ Level

**Figure 1:**
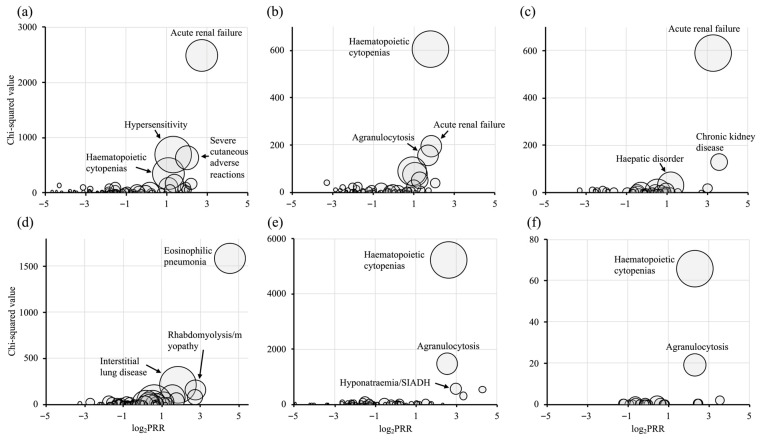
Volcano plots based on narrow SMQ codes. (**a**): vancomycin; (**b**): teicoplanin; (**c**): arbekacin; (**d**): daptomycin; (**e**): linezolid; (**f**): tedizolid. The vertical axis represents the chi-squared value, the horizontal axis shows the base-2 logarithm of the proportional reporting ratio (PRR), and the plot size corresponds to the reporting ratio.

The SMQ-level signal detection results are presented in Table 2 and Figure 1. Eleven SMQs met the signal detection criteria for VCM, with the highest signals observed for acute renal failure (PRR = 6.66), hypersensitivity (PRR = 2.50), and severe cutaneous adverse reactions (PRR = 4.03). The TEIC revealed eight SMQs, notably haematopoietic cytopenias (PRR = 3.51), acute renal failure (PRR = 3.61), and agranulocytosis (PRR = 3.26). ABK was associated with four SMQs: acute renal failure (PRR = 9.75) and chronic kidney disease (PRR = 11.82). Among the DAP-related AEs, eight SMQs were identified: eosinophilic pneumonia (PRR = 23.30), interstitial lung disease (PRR = 3.56), and rhabdomyolysis/myopathy (PRR = 6.71). LZD signals were dominated by haematopoietic cytopenias and agranulocytosis, with LZD haematopoietic cytopenias reaching a PRR of 6.13. Furthermore, LZD was frequently reported in conjunction with hyponatremia/syndrome of inappropriate antidiuretic hormone secretion (SIADH), optic neuropathy, and lactic acidosis, indicating a broad spectrum of reported adverse events. In contrast, TZD was associated with a limited number of reports (*n* = 39) in the JADER database. Although haematopoietic cytopenias and agranulocytosis were observed, these findings should be interpreted with caution due to the small number of cases and potential statistical variability. These findings suggest that each agent exhibits distinct AE profiles, with certain event categories showing disproportionate reporting patterns within individual drugs. Rather than implying quantitative comparisons of risk between agents, the SMQ-level analysis was intended to provide a qualitative overview of the types of AEs characteristically reported for each drug. Sensitivity analyses using the ROR and IC_025_ yielded results broadly consistent with those of the primary PRR analysis. Most signals identified by PRR were also detected by both ROR and IC, supporting the robustness of the findings.

### 3.3. Signal Detection at the PT Level

**Figure 2:**
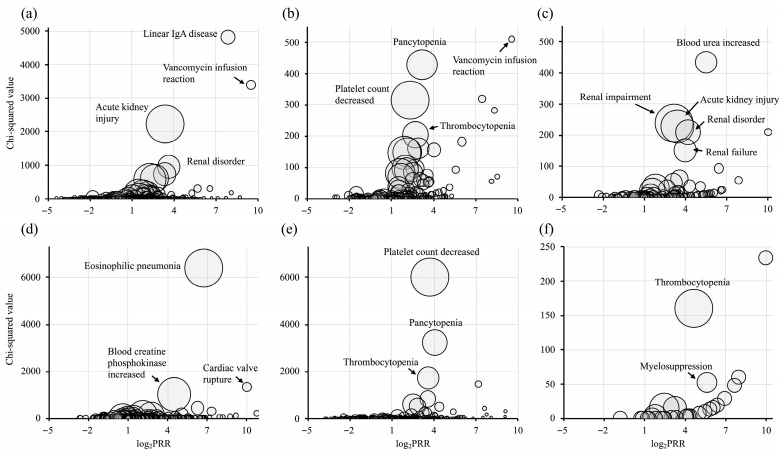
Volcano plots based on PT codes. (**a**): vancomycin; (**b**): teicoplanin; (**c**): arbekacin; (**d**): daptomycin; (**e**): linezolid; (**f**): tedizolid. The vertical axis represents the chi-squared value, the horizontal axis shows the base-2 logarithm of the proportional reporting ratio (PRR), and the plot size corresponds to the reporting ratio.

The PT-level analysis summarized in Figure 2 and Supplementary Table S1 provides more granular, descriptive characterization of the AE profiles. For VCM, sixty-two PTs met the signal detection criteria, including linear Immunoglobulin A (IgA) disease (PRR = 229.12), VCM infusion reaction (PRR = 719.37), and acute kidney injury (PRR = 10.44). TEIC was associated with forty-one PTs, predominantly pancytopenia (PRR = 9.04), decreased platelet count (PRR = 5.00), and infusion reactions (PRR = 179.82). ABK-related signals encompassed 17 PTs with increased blood urea (PRR = 45.86) and acute kidney injury (PRR = 10.64) being most notable. The DAP signals included eosinophilic pneumonia (PRR = 106.18) and increased blood creatine phosphokinase (CPK) levels (PRR = 22.59). LZD and TZD signals primarily reflected hematological toxicity, including thrombocytopenia (LZD, PRR = 12.09; TZD, PRR = 24.76) and pancytopenia. All PTs that met the PRR-based signal detection criteria also fulfilled the ROR criteria. However, a small number of PTs identified by PRR were not detected by IC_025_ (e.g., VCM [*n* = 2], TEIC [*n* = 1], ABK [*n* = 1], DAP [*n* = 2], and LZD [*n* = 1]), suggesting potential instability due to sparse data. This analysis further clarifies the specific events underlying the SMQ-level signals and describes drug-specific reporting patterns, without implying causal relationships or quantitative differences in risk across agents.

### 3.4. Time to Onset and Weibull Distribution Analysis of AEs at the SMQ Level

**Figure 3:**
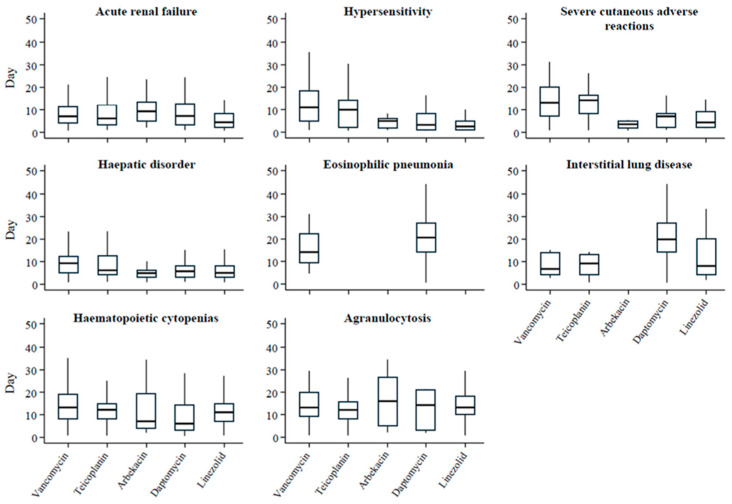
Box plots of time-to-onset distributions for major SMQs. The vertical axis indicates the number of days from treatment initiation.

Table 3 presents the results of the Weibull distribution analysis at the SMQ level. Only SMQs with a sufficient number of reports and available start and end dates of administration, including acute renal failure, hypersensitivity, severe cutaneous adverse reactions, hepatic disorders, eosinophilic pneumonia, interstitial lung disease, haematopoietic cytopenias, and agranulocytosis, were included. Figure 3 illustrates the distribution of time to onset for the major SMQs using box plots, providing an overview of their temporal occurrence patterns.

For VCM, TEIC, and ABK, acute renal failure, severe cutaneous adverse reactions, hepatic disorders, haematopoietic cytopenias, and agranulocytosis generally exhibited wear-out failure patterns (β > 1), suggesting a tendency for increased reporting with longer exposure, possibly reflecting a cumulative risk of these adverse events. Hypersensitivity and interstitial lung disease were classified as random failures (β ≈ 1), while eosinophilic pneumonia had insufficient cases for robust analysis. For DAP, hepatic disorders, eosinophilic pneumonia, and interstitial lung disease were wear-out failures, whereas acute renal failure, hypersensitivity, severe cutaneous adverse reactions, haematopoietic cytopenias, and agranulocytosis were random failures. For LZD, only haematopoietic cytopenias showed a wear-out pattern, whereas other AEs, including acute renal failure, hypersensitivity, severe cutaneous adverse reactions, hepatic disorders, interstitial lung disease, and agranulocytosis, were random failures. Eosinophilic pneumonia was not analyzed. TZDs were excluded from the analysis owing to the limited number of reports (*n* = 39).

Although the AD test indicated statistically significant deviations from the Weibull distribution in several drug–AE combinations, the Weibull model was used as a descriptive approximation of the observed time-to-onset patterns rather than as a strict parametric fit.

Overall, the estimated Weibull shape parameters suggested that renal, hepatic, and hematopoietic AEs tended to occur more frequently with prolonged exposure to most anti-MRSA agents, whereas hypersensitivity and pulmonary AEs generally occurred randomly over time.

## 4. Discussion

In this nationwide pharmacovigilance study using the JADER database over two decades, the safety profiles of six anti-MRSA agents (VCM, TEIC, ABK, DAP, LZD, and TZD) were characterized using disproportionality analyses at both the SMQ and PT levels, complemented by Weibull-based time-to-onset modeling. Three main observations emerged: First, each drug showed a distinct pattern of reported AEs that was broadly consistent with its pharmacology and previous literature, including nephrotoxicity for VCM and ABK, hematological toxicity for LZD, and pulmonary/muscle-related events for DAP. Second, Weibull analyses indicated “wear-out” hazards (β > 1) for renal, hepatic, and hematologic events, suggesting a tendency toward later occurrence with prolonged exposure, whereas hypersensitivity and some pulmonary events showed “random-failure” patterns (β ≈ 1), indicating no clear temporal clustering. Third, the combined SMQ- and PT-level approach helped to descriptively delineate clinically recognizable AE phenotypes, such as DAP-related eosinophilic pneumonia, CPK elevation, and VCM-associated infusion reactions, which may be relevant for routine clinical monitoring.

Among the key observations was a disproportionate reporting pattern for eosinophilic pneumonia reported for DAP (SMQ-level PRR ~23), along with elevated CPK and rhabdomyolysis signals at the PT level. These results are in line with multiple reviews and pharmacovigilance analyses indicating that DAP is frequently reported in association with drug-induced eosinophilic pneumonia, typically occurring in older males after prolonged exposure, often beyond 2–3 weeks [17,18,19]. Recent FAERS-based studies similarly reported a higher reporting frequency of DAP-related eosinophilic pneumonia compared to other anti-MRSA agents and suggested a possible duration-dependent tendency [17]. Case series and systematic reviews have described a characteristic symptom constellation, including fever, dyspnea, and hypoxemia, accompanied by bilateral radiographic infiltrates and rapid improvement after DAP discontinuation and corticosteroids [18,19]. The Weibull analysis demonstrated wear-out features (β > 1) for both eosinophilic pneumonia and interstitial lung disease, reinforcing the need for heightened pulmonary vigilance during ongoing treatment. Together with the established myopathy and rhabdomyolysis signals, these findings may be relevant for clinical awareness and monitoring of pulmonary and muscle-related events in patients receiving DAP, particularly in older adults and during extended treatment courses.

As anticipated, LZD demonstrated disproportionate reporting of haematopoietic cytopenia (PRR > 6), agranulocytosis, hyponatremia/SIADH, lactic acidosis, and optic neuropathy. These are well aligned with prior observational studies and pharmacovigilance reports linking prolonged LZD therapy to marrow suppression and mitochondrial toxicity [20,21,22,23]. The wear-out hazard observed for hematologic events mirrors clinical patterns, where cytopenia typically emerges within 1–2 weeks [20,21]. Optic neuropathy and lactic acidosis signals, which are consistent with mitochondrial dysfunction, may support the need for careful ophthalmological and metabolic monitoring in prolonged use [22,23].

Our analysis also illustrated differences in the reported renal safety patterns of glycopeptides and aminoglycosides. VCM showed disproportionate reporting of acute renal failure and tubulointerstitial diseases (PRR 4–7), consistent with previously reported nephrotoxicity patterns [24]. The wear-out pattern is consistent with a cumulative reporting pattern and justifies the Area under the curve-guided therapeutic monitoring [25]. In contrast, TEIC exhibited fewer renal signals but relatively more hematological associations, consistent with reports of lower nephrotoxicity but an ongoing cytopenia risk during extended use [5,26]. ABK, primarily used in Japan, shows high PRRs for acute and chronic renal failure, reflecting well-recognized class effects of aminoglycosides [6]. Although ototoxicity was less apparent in the data set, the clinical experience suggests that it is a relevant concern. At the cutaneous level, VCM displays expected signals for severe cutaneous adverse reactions and unique entities such as linear IgA bullous dermatosis and infusion-related reactions [27]. The Weibull results indicated wear-out features for severe cutaneous reactions, consistent with delayed-type immune mechanisms, whereas hypersensitivity showed random failure characteristics, indicating the need for vigilance throughout treatment.

The findings for TZD should be interpreted with caution due to the small number of reports (*n* = 39) in the JADER database. Although some hematological signals were observed, these findings are exploratory and potentially underpowered, and no firm conclusions can be drawn. TZD was also excluded from the Weibull analysis because of the limited number of cases with complete time-to-onset data, further limiting the assessment of temporal patterns. These limitations highlight the need for evaluation in larger datasets.

From a clinical perspective, our results may inform monitoring strategies for anti-MRSA agents. For daptomycin, monitoring of CPK may be relevant, while for linezolid, regular complete blood count monitoring may help detect hematological adverse events. The Weibull analysis suggests that renal, hepatic, and hematological adverse events may be more frequently reported with increasing treatment duration, indicating the need for closer monitoring during prolonged therapy, whereas hypersensitivity and pulmonary adverse events may occur unpredictably, suggesting the need for continuous vigilance throughout the treatment course. Although these findings are based on reporting data, they may provide useful guidance for clinical practice.

This study has several limitations inherent to SRSs. First, disproportionality measures such as the PRR reflect reporting frequency rather than incidence or absolute risk and do not establish causal relationships. In addition, the JADER database lacks denominator data, preventing the estimation of incidence rates and precluding direct quantitative comparisons between drugs, and is subject to underreporting and reporting bias, including potential temporal trends such as the Weber effect. Second, unmeasured confounding factors, including renal function, comorbidities, and disease severity, could not be fully accounted for. In particular, indication bias may exist, as certain agents (e.g., DAP or LZD) may be preferentially used in more severe or refractory cases. Third, the analysis was restricted to drugs designated as “suspected,” which may introduce channeling bias and reflect differential drug utilization patterns in clinical practice. Fourth, incomplete or inconsistent time data may have led to selection bias in the time-to-onset analysis, and time-dependent reporting bias cannot be excluded. In addition, the use of a uniform 14-day post-discontinuation window to define adverse events may introduce potential misclassification. The latency of adverse events varies depending on the event type; some occur shortly after discontinuation, whereas others may have delayed onset. Therefore, this fixed time window may result in differential misclassification across adverse event types and should be considered when interpreting the findings. Fifth, important clinical variables, including dosage, treatment duration, serum concentrations, and renal function, were not available, limiting detailed risk assessment. Furthermore, differences in clinical use and administration routes across anti-MRSA agents may introduce heterogeneity in patient populations and influence adverse event profiles. In addition, patients are often exposed to multiple concomitant medications, including nephrotoxic and myelosuppressive agents, which cannot be adequately adjusted for in the JADER database. Therefore, particularly for nephrotoxicity and cytopenias, observed signals may be partly attributable to polypharmacy. Finally, as the JADER database primarily reflects reports from Japanese patients, the generalizability of these findings to other populations remains uncertain. Overall, the results should be interpreted as within-drug reporting patterns rather than direct comparative risk estimates across agents.

Despite these constraints, the concordance of our findings with prior clinical observations may support the clinical plausibility of several well-recognized associations, particularly DAP-related pulmonary toxicity, LZD-associated hematologic events, and VCM nephrotoxicity. Such consistency suggests that JADER-based analyses can offer complementary, hypothesis-generating insights, which may inform future observational or mechanistic studies.

## 5. Conclusions

This large-scale JADER analysis characterized agent-specific patterns of reported AEs among six anti-MRSA drugs. Frequently reported signals include DAP (eosinophilic pneumonia and myopathy), LZD (hematologic and mitochondrial toxicities), and VCM (nephrotoxicity and cutaneous reactions). Time-to-onset modeling suggested a tendency toward later occurrence for renal, hepatic, and hematologic events, whereas hypersensitivity and many pulmonary events showed no clear temporal clustering. Rather than providing quantitative risk estimates, these findings descriptively indicate clinically recognizable safety patterns that may support tailored monitoring strategies. Future studies using multiple databases and patient-level designs are warranted to better quantify these risks and guide the safer use of anti-MRSA agents.

## Figures and Tables

**Table 1:** Demographic characteristics of adverse event reports associated with anti-MRSA agents in the JADER database.

	Case (%)					
Characteristics	Vancomycin	Teicoplanin	Arbekacin	Daptomycin	Linezolid	Tedizolid
Total case	2318	838	227	552	1750	39
Sex						
Male	1409 (60.8%)	511 (61.0%)	153 (67.4%)	379 (68.7%)	1069 (61.1%)	26 (66.7%)
Female	892 (38.5%)	306 (36.5%)	70 (30.8%)	159 (28.8%)	608 (34.7%)	11 (28.2%)
not specified	17 (0.7%)	21 (2.5%)	4 (1.8%)	14 (2.5%)	73 (4.2%)	2 (5.1%)
Age [years]						
~19	146 (6.3%)	70 (8.4%)	10 (4.4%)	21 (3.8%)	43 (2.5%)	1 (2.6%)
20~59	690 (29.8%)	214 (25.5%)	31 (13.7%)	137 (24.8%)	326 (18.6%)	8 (20.5%)
60+	1392 (60.1%)	525 (62.6%)	183 (80.6%)	384 (69.6%)	1301 (74.3%)	24 (61.5%)
not specified	90 (3.9%)	29 (3.5%)	3 (1.3%)	10 (1.8%)	80 (4.6%)	6 (15.4%)
Body weight [kg]						
~39	195 (8.4%)	81 (9.7%)	26 (11.5%)	19 (3.4%)	132 (7.5%)	2 (5.1%)
40~69	1089 (47.0%)	381 (45.5%)	126 (55.5%)	250 (45.3%)	667 (38.1%)	12 (30.8%)
70+	218 (9.4%)	70 (8.4%)	13 (5.7%)	89 (16.1%)	95 (5.4%)	3 (7.7%)
not specified	816 (35.2%)	306 (36.5%)	62 (27.3%)	194 (35.1%)	856 (48.9%)	22 (56.4%)

**Table 2:** SMQs with detected signals for anti-MRSA agents.

Drug	SMQ	With the Target Drug	Without the Target Drug	PRR	Chi-Squared Value	ROR [95% CI]	IC_025_	log FDR-Adjusted *p*-Value
		AE Cases/Total Cases (%)	AE Cases/Total Cases (%)					
Vancomycin	Acute renal failure	510/2318 (22%)	31,495/952,859 (3.31%)	6.66	2490.21	8.25 [7.85–8.68]	2.58	−1244.90
	Hypersensitivity	684/2318 (29.51%)	112,677/952,859 (11.83%)	2.50	689.60	3.12 [2.98–3.27]	1.21	−344.64
	Severe cutaneous adverse reactions	273/2318 (11.78%)	27,823/952,859 (2.92%)	4.03	632.35	4.44 [4.16–4.74]	1.83	−316.38
	Haematopoietic cytopenias	513/2318 (22.13%)	98,672/952,859 (10.36%)	2.14	343.32	2.46 [2.34–2.59]	0.97	−171.85
	Oropharyngeal disorders	165/2318 (7.12%)	25,951/952,859 (2.72%)	2.61	166.28	2.74 [2.52–2.97]	1.17	−83.20
	Tubulointerstitial diseases	59/2318 (2.55%)	5258/952,859 (0.55%)	4.61	162.42	4.71 [4.12–5.37]	1.78	−81.44
	Agranulocytosis	176/2318 (7.59%)	34,207/952,859 (3.59%)	2.12	105.62	2.21 [2.04–2.39]	0.88	−53.11
	Chronic kidney disease	46/2318 (1.98%)	4950/952,859 (0.52%)	3.82	92.59	3.88 [3.34–4.5]	1.47	−46.65
	Drug reaction with eosinophilia and systemic symptoms syndrome	44/2318 (1.90%)	5152/952,859 (0.54%)	3.51	76.27	3.56 [3.05–4.15]	1.35	−38.68
	Hearing and vestibular disorders	27/2318 (1.16%)	2878/952,859 (0.30%)	3.86	53.96	3.89 [3.2–4.73]	1.31	−27.58
	Pseudomembranous colitis	21/2318 (0.91%)	2085/952,859 (0.22%)	4.14	46.55	4.17 [3.34–5.2]	1.30	−23.93
	Sepsis	59/2318 (2.55%)	10,684/952,859 (1.12%)	2.27	40.90	2.3 [2.02–2.63]	0.82	−21.15
Teicoplanin	Haematopoietic cytopenias	305/838 (36.40%)	98,880/954,339 (10.36%)	3.51	607.07	4.95 [4.61–5.32]	1.64	−302.89
	Acute renal failure	101/838 (12.05%)	31,904/954,339 (3.34%)	3.61	193.44	3.96 [3.56–4.41]	1.55	−96.20
	Agranulocytosis	98/838 (11.69%)	34,285/954,339 (3.59%)	3.26	156.06	3.55 [3.19–3.96]	1.41	−77.81
	Hepatic disorders	130/838 (15.51%)	71,835/954,339 (7.53%)	2.06	75.50	2.26 [2.05–2.48]	0.80	−37.69
	Severe cutaneous adverse reactions	60/838 (7.16%)	28,036/954,339 (2.94%)	2.44	50.81	2.55 [2.23–2.91]	0.92	−25.33
	Chronic kidney disease	18/838 (2.15%)	4978/954,339 (0.52%)	4.12	39.49	4.19 [3.3–5.31]	1.22	−19.84
	Acute pancreatitis	11/838 (1.31%)	4881/954,339 (0.51%)	2.57	9.03	2.59 [1.91–3.51]	0.41	−4.63
	Pseudomembranous colitis	6/838 (0.72%)	2100/954,339 (0.22%)	3.25	7.24	3.27 [2.17–4.93]	0.26	−3.86
Arbekacin	Acute renal failure	74/227 (32.60%)	31,931/954,950 (3.34%)	9.75	590.79	13.98 [12.13–16.11]	2.79	−295.03
	Chronic kidney disease	14/227 (6.17%)	4982/954,950 (0.52%)	11.82	128.39	12.53 [9.51–16.52]	2.09	−63.76
	Hepatic disorders	39/227 (17.18%)	71,926/954,950 (7.53%)	2.28	28.96	2.55 [2.14–3.04]	0.71	−13.73
	Pseudomembranous colitis	4/227 (1.76%)	2102/954,950 (0.22%)	8.01	18.02	8.13 [4.91–13.47]	0.46	−8.33
Daptomycin	Eosinophilic pneumonia	76/552 (13.77%)	5641/954,625 (0.59%)	23.30	1588.04	26.86 [23.72–30.41]	3.86	−793.86
	Interstitial lung disease	111/552 (20.11%)	53,977/954,625 (5.65%)	3.56	213.07	4.2 [3.78–4.67]	1.54	−106.06
	Rhabdomyolysis/myopathy	33/552 (5.98%)	8499/954,625 (0.89%)	6.71	155.63	7.08 [5.91–8.47]	2.07	−77.59
	Lack of efficacy/effect	17/552 (3.08%)	4441/954,625 (0.47%)	6.62	75.65	6.8 [5.31–8.7]	1.71	−37.53
	Acute renal failure	54/552 (9.78%)	31,951/954,625 (3.35%)	2.92	68.58	3.13 [2.71–3.61]	1.14	−34.18
	Sepsis	21/552 (3.80%)	10,722/954,625 (1.12%)	3.39	33.29	3.48 [2.79–4.35]	1.05	−16.37
	Severe cutaneous adverse reactions	35/552 (6.34%)	28,061/954,625 (2.94%)	2.16	21.18	2.24 [1.88–2.66]	0.63	−10.50
	Acute central respiratory depression	10/552 (1.81%)	7048/954,625 (0.74%)	2.45	7.26	2.48 [1.8–3.41]	0.31	−3.59
Linezolid	Haematopoietic cytopenias	1103/1750 (63.03%)	98,082/953,427 (10.29%)	6.13	5215.83	14.87 [14.15–15.62]	2.50	−2608.12
	Agranulocytosis	361/1750 (20.63%)	34,022/953,427 (3.57%)	5.78	1460.20	7.02 [6.62–7.45]	2.36	−730.36
	Hyponatremia/SIADH	96/1750 (5.49%)	6745/953,427 (0.71%)	7.75	554.18	8.15 [7.33–9.05]	2.58	−277.27
	Optic nerve disorders	33/1750 (1.89%)	931/953,427 (0.10%)	19.31	536.34	19.66 [16.44–23.51]	3.18	−268.62
	Lactic acidosis	39/1750 (2.23%)	2088/953,427 (0.22%)	10.18	308.51	10.39 [8.82–12.23]	2.63	−154.66
	Peripheral neuropathy	38/1750 (2.17%)	7446/953,427 (0.78%)	2.78	41.67	2.82 [2.39–3.32]	1.00	−21.28
	Pseudomembranous colitis	13/1750 (0.74%)	2093/953,427 (0.22%)	3.38	19.43	3.4 [2.57–4.5]	0.82	−10.16
	Torsade de pointes/QT prolongation	22/1750 (1.26%)	5832/953,427 (0.61%)	2.06	10.91	2.07 [1.67–2.56]	0.44	−5.98
	Taste and smell disorders	7/1750 (0.40%)	1159/953,427 (0.12%)	3.29	8.94	3.3 [2.26–4.82]	0.39	−5.01
	Lack of efficacy/effect	17/1750 (0.97%)	4441/953,427 (0.47%)	2.09	8.56	2.1 [1.64–2.68]	0.36	−4.83
	Hearing and vestibular disorders	12/1750 (0.69%)	2893/953,427 (0.30%)	2.26	7.21	2.27 [1.7–3.03]	0.31	−4.28
Tedizolid *^1^	Haematopoietic cytopenias	20/39 (51.28%)	99,165/955,138 (10.38%)	4.94	65.78	9.09 [6.6–12.52]	1.36	−32.13
	Agranulocytosis	7/39 (17.95%)	34,376/955,138 (3.60%)	4.99	19.19	5.86 [3.86–8.89]	0.69	−8.95

*^1^: Signals for TZD are based on a small number of reports (*n* = 39) and should be interpreted cautiously. These findings are exploratory and may be influenced by statistical variability. AE, adverse event; CI, confidence interval; FDR, false discovery rate; IC_025_, lower bound of the 95% credibility interval for information component; PRR, proportional reporting ratio; ROR, reporting odds ratio; SIADH, Syndrome of inappropriate secretion of antidiuretic hormone; SMQ, Standardized MedDRA^®^ Query.

**Table 3:** Results of Weibull distribution analysis based on SMQ codes.

SMQ		Vancomycin	Teicoplanin	Arbekacin	Daptomycin	Linezolid
Acute renal failure	number of reports	212	62	61	27	29
	Median [IQR]	7 [4–11.2]	6 [3–12]	9 [5–13]	7 [3–12.5]	4 [2–8]
	α [95% CI]	10.04 [8.98–11.11]	10.08 [7.94–12.21]	10.21 [8.67–11.76]	10.91 [7.35–14.46]	7.6 [4.17–11.03]
	β [95% CI]	1.35 [1.22–1.48]	1.25 [1.02–1.47]	1.75 [1.40–2.10]	1.23 [0.88–1.57]	0.86 [0.63–1.08]
	Type	wear-out failure	wear-out failure	wear-out failure	random failure	random failure
	P (AD test)	0.044	0.273	0.615	0.824	0.392
Hypersensitivity	number of reports	387	128	15	74	36
	Median [IQR]	11 [5–18]	10 [2–14]	5 [1.5–6]	3 [1–8]	3 [1–5]
	α [95% CI]	13.87 [12.53–15.21]	11.6 [9.29–13.91]	NA	5.12 [3.85–6.39]	4.2 [2.96–5.44]
	β [95% CI]	1.09 [1.0049–1.17]	0.92 [0.81–1.03]	NA	0.98 [0.81–1.14]	1.17 [0.89–1.45]
	Type	wear-out failure	random failure	NA	random failure	random failure
	P (AD test)	0.003 ^†^	0.028	NA	0.022	0.234
Severe cutaneous adverse reactions	number of reports	156	45	8	30	8
	Median [IQR]	13 [7–20]	14 [8–16.2]	3.5 [1.75–5]	7 [2–8]	4 [2–9]
	α [95% CI]	16.68 [14.34–19.01]	15.85 [12.93–18.76]	NA	6.91 [4.99–8.83]	NA
	β [95% CI]	1.2 [1.06–1.34]	1.68 [1.29–2.08]	NA	1.38 [0.98–1.79]	NA
	Type	wear-out failure	wear-out failure	NA	random failure	NA
	P (AD test)	0.090	0.532	NA	0.452	NA
Hepatic disorders	number of reports	108	91	29	44	39
	Median [IQR]	9 [5–12.5]	6 [4–12.5]	5 [3–6]	5.5 [3–8]	5 [3–8]
	α [95% CI]	10.67 [9.12–12.21]	9.26 [7.90–10.61]	6.61 [5.04–8.18]	7.81 [6.25–9.37]	8.01 [5.23–10.78]
	β [95% CI]	1.37 [1.17–1.57]	1.48 [1.25–1.72]	1.63 [1.21–2.05]	1.57 [1.22–1.91]	0.96 [0.76–1.17]
	Type	wear-out failure	wear-out failure	wear-out failure	wear-out failure	random failure
	P (AD test)	0.625	0.132	0.139	0.609	0.133
Eosinophilic pneumonia	number of reports	3	NA	NA	56	NA
	Median [IQR]	14 [9.5–22.5]	NA	NA	20.5 [14–27]	NA
	α [95% CI]	NA	NA	NA	25.15 [20.95–29.35]	NA
	β [95% CI]	NA	NA	NA	1.65 [1.34–1.97]	NA
	Type	NA	NA	NA	wear-out failure	NA
	P (AD test)	NA	NA	NA	0.296	NA
Interstitial lung disease	number of reports	16	12	2	77	11
	Median [IQR]	6.5 [4–14.2]	9 [4.25–13.2]	7.5 [7.25–7.75]	20 [14–27]	8 [4–20]
	α [95% CI]	NA	NA	NA	23.99 [21.00–26.97]	NA
	β [95% CI]	NA	NA	NA	1.89 [1.58–2.20]	NA
	Type	NA	NA	NA	wear-out failure	NA
	P (AD test)	NA	NA	NA	0.389	NA
Haematopoietic cytopenias	number of reports	310	193	22	33	492
	Median [IQR]	13 [8–19]	12 [8–15]	7 [4–19]	6 [3–14]	11 [7–15]
	α [95% CI]	15.87 [14.73–17.01]	13.74 [12.60–14.87]	12.73 [8.19–17.27]	12.16 [7.38–16.94]	13.97 [12.9–15.04]
	β [95% CI]	1.63 [1.49–1.77]	1.79 [1.60–1.98]	1.24 [0.84–1.64]	0.92 [0.70–1.15]	1.22 [1.15–1.29]
	Type	wear-out failure	wear-out failure	random failure	random failure	wear-out failure
	P (AD test)	0.485	0.100	0.625	0.446	1.22 × 10^−6 †^
Agranulocytosis	number of reports	89	47	7	9	128
	Median [IQR]	13 [9–20]	12 [8–15.5]	16 [5–26.5]	14 [3–21]	13 [9.75–18]
	α [95% CI]	16.68 [14.58–18.79]	13.96 [11.73–16.18]	NA	NA	18.23 [15.21–21.25]
	β [95% CI]	1.73 [1.45–2.02]	1.87 [1.44–2.31]	NA	NA	1.11 [0.99–1.23]
	Type	wear-out failure	wear-out failure	NA	NA	random failure
	P (AD test)	0.902	0.562	NA	NA	2.54 × 10^−5 †^

AD, Anderson-Daring; CI, confidence interval; IQR, interquartile range; NA, not applicable; SMQ, Standardized MedDRA^®^ Query; NA, not applicable; α, scale parameter; β, shape parameter; ^†^, Anderson–Darling test indicates poor Weibull fit (*p* < 0.01); classification should be interpreted as descriptive only.

## Data Availability

The datasets used and/or analysed during the current study are available from the corresponding author on reasonable request.

## Supplementary Materials

The following supporting information can be downloaded at: https://www.mdpi.com/article/10.3390/idr18030043/s1. Table S1. PTs with detected signals for anti-MRSA agents.
