# Supplementary material for: A Real-World Pharmacovigilance Analysis of the Safety Profiles Associated with Anti-MRSA Agents Using the Japanese Adverse Drug Event Report (JADER) Database
Source: Infect Dis Rep. 2026 May 2;18(3):43. doi: 10.3390/idr18030043 (PMC13214683; doi:10.3390/idr18030043)
Supplement: Supplementary file 1 [file idr-18-00043-s001.zip › idr-4201257-supplementary.docx]

**Supplementary Table S1.** PTs with detected signals for anti-MRSA agents.

| Drug | PT code | PT | with target drug | without target drug | PRR | Chi-squared value | ROR  [95%CI] | IC_025_ | log FDR-adjusted p-value |
| --- | --- | --- | --- | --- | --- | --- | --- | --- | --- |
|  |  |  | AE cases/total cases (%) | AE cases/total cases (%) |  |  |  |  |  |
| Vancomycin | 1002451 | Linear IgA disease | 34/2,318 (1.47%) | 61/952,859 (0.01%) | 229.12 | 4,813.23 | 232.52 [187.53-288.3] | 4.34 | -2404.83 |
|  | 1008673 | Vancomycin infusion reaction | 14/2,318 (0.60%) | 8/952,859 (<0.01%) | 719.37 | 3,394.99 | 723.74 [464.39-1127.91] | 3.01 | -1696.23 |
|  | 1006933 | Acute kidney injury | 265/2,318 (11.43%) | 10,436/952,859 (1.10%) | 10.44 | 2,221.18 | 11.66 [10.91-12.45] | 3.14 | -1109.52 |
|  | 1003842 | Renal disorder | 95/2,318 (4.10%) | 3,174/952,859 (0.33%) | 12.30 | 950.17 | 12.79 [11.5-14.22] | 3.17 | -473.88 |
|  | 1004422 | Toxic epidermal necrolysis | 93/2,318 (4.01%) | 3,796/952,859 (0.40%) | 10.07 | 735.81 | 10.45 [9.39-11.63] | 2.91 | -366.80 |
|  | 1001368 | Drug eruption | 145/2,318 (6.26%) | 9,840/952,859 (1.03%) | 6.06 | 604.71 | 6.39 [5.87-6.97] | 2.32 | -301.33 |
|  | 1006223 | Renal impairment | 192/2,318 (8.28%) | 16,534/952,859 (1.74%) | 4.77 | 572.45 | 5.11 [4.74-5.52] | 2.03 | -285.33 |
|  | 1004256 | Superinfection | 8/2,318 (0.35%) | 65/952,859 (0.01%) | 50.59 | 303.46 | 50.76 [34.88-73.88] | 2.00 | -150.65 |
|  | 1004915 | Neutropenic sepsis | 5/2,318 (0.22%) | 22/952,859 (<0.01%) | 93.42 | 300.86 | 93.62 [57.02-153.72] | 1.28 | -149.46 |
|  | 1003366 | Pancytopenia | 81/2,318 (3.49%) | 7,668/952,859 (0.80%) | 4.34 | 204.56 | 4.46 [3.98-5] | 1.77 | -101.22 |
|  | 1003784 | Rash | 114/2,318 (4.92%) | 13,494/952,859 (1.42%) | 3.47 | 199.43 | 3.6 [3.27-3.97] | 1.52 | -98.75 |
|  | 1005361 | Type IV hypersensitivity reaction | 6/2,318 (0.26%) | 64/952,859 (0.01%) | 38.54 | 167.66 | 38.64 [25.2-59.24] | 1.51 | -82.94 |
|  | 1003552 | Platelet count decreased | 148/2,318 (6.38%) | 22,270/952,859 (2.34%) | 2.73 | 163.53 | 2.85 [2.62-3.1] | 1.22 | -80.94 |
|  | 1007158 | Haemophagocytic lymphohistiocytosis | 28/2,318 (1.21%) | 1,539/952,859 (0.16%) | 7.48 | 148.27 | 7.56 [6.24-9.16] | 2.12 | -73.33 |
|  | 1000150 | Agranulocytosis | 52/2,318 (2.24%) | 4,532/952,859 (0.48%) | 4.72 | 147.61 | 4.8 [4.17-5.53] | 1.78 | -73.06 |
|  | 1003843 | Renal failure | 42/2,318 (1.81%) | 3,173/952,859 (0.33%) | 5.44 | 146.39 | 5.52 [4.72-6.46] | 1.90 | -72.51 |
|  | 1000548 | Blood creatinine increased | 35/2,318 (1.51%) | 2,456/952,859 (0.26%) | 5.86 | 134.62 | 5.93 [5-7.04] | 1.93 | -66.64 |
|  | 1004830 | Tubulointerstitial nephritis | 40/2,318 (1.73%) | 3,193/952,859 (0.34%) | 5.15 | 128.46 | 5.22 [4.45-6.13] | 1.82 | -63.59 |
|  | 1004203 | Stevens-Johnson syndrome | 63/2,318 (2.72%) | 6,810/952,859 (0.71%) | 3.80 | 127.10 | 3.88 [3.41-4.41] | 1.54 | -62.95 |
|  | 1004794 | White blood cell count decreased | 100/2,318 (4.31%) | 14,381/952,859 (1.51%) | 2.86 | 119.97 | 2.94 [2.66-3.26] | 1.23 | -59.41 |
|  | 1001515 | Erythema | 53/2,318 (2.29%) | 6,181/952,859 (0.65%) | 3.52 | 93.15 | 3.58 [3.12-4.12] | 1.40 | -45.92 |
|  | 1003854 | Renal tubular necrosis | 9/2,318 (0.39%) | 264/952,859 (0.03%) | 14.01 | 92.97 | 14.06 [10.01-19.75] | 1.73 | -45.87 |
|  | 1001494 | Eosinophil count increased | 16/2,318 (0.69%) | 891/952,859 (0.09%) | 7.38 | 80.62 | 7.43 [5.77-9.56] | 1.77 | -39.67 |
|  | 1000585 | Blood urea increased | 17/2,318 (0.73%) | 1,003/952,859 (0.11%) | 6.97 | 79.74 | 7.01 [5.49-8.96] | 1.76 | -39.27 |
|  | 1007350 | Drug reaction with eosinophilia and systemic symptoms | 44/2,318 (1.90%) | 5,141/952,859 (0.54%) | 3.52 | 76.57 | 3.57 [3.06-4.16] | 1.35 | -37.70 |
|  | 1001187 | Deafness | 15/2,318 (0.65%) | 876/952,859 (0.09%) | 7.04 | 70.64 | 7.08 [5.45-9.19] | 1.68 | -34.73 |
|  | 1003766 | Pyrexia | 108/2,318 (4.66%) | 21,756/952,859 (2.28%) | 2.04 | 57.31 | 2.09 [1.89-2.31] | 0.77 | -28.00 |
|  | 1003853 | Renal tubular disorder | 8/2,318 (0.35%) | 354/952,859 (0.04%) | 9.29 | 50.05 | 9.32 [6.51-13.33] | 1.35 | -24.50 |
|  | 1000377 | Auditory disorder | 3/2,318 (0.13%) | 44/952,859 (<0.01%) | 28.03 | 50.03 | 28.06 [15.45-50.98] | 0.36 | -24.50 |
|  | 1001245 | Dermatitis exfoliative generalised | 12/2,318 (0.52%) | 760/952,859 (0.08%) | 6.49 | 49.62 | 6.52 [4.87-8.73] | 1.44 | -24.32 |
|  | 1002075 | Hypersensitivity | 24/2,318 (1.04%) | 2,513/952,859 (0.26%) | 3.93 | 49.10 | 3.96 [3.22-4.86] | 1.29 | -24.08 |
|  | 1005797 | Toxic skin eruption | 20/2,318 (0.86%) | 1,901/952,859 (0.20%) | 4.32 | 47.44 | 4.35 [3.47-5.46] | 1.33 | -23.26 |
|  | 1007736 | Multiple organ dysfunction syndrome | 21/2,318 (0.91%) | 2,084/952,859 (0.22%) | 4.14 | 46.59 | 4.17 [3.35-5.2] | 1.30 | -22.85 |
|  | 1004006 | Septic embolus | 3/2,318 (0.13%) | 49/952,859 (0.01%) | 25.17 | 44.76 | 25.2 [13.9-45.69] | 0.34 | -21.95 |
|  | 1004763 | Vitamin K deficiency | 4/2,318 (0.17%) | 111/952,859 (0.01%) | 14.81 | 37.27 | 14.84 [8.92-24.69] | 0.68 | -18.16 |
|  | 1003712 | Pseudomembranous colitis | 13/2,318 (0.56%) | 1,161/952,859 (0.12%) | 4.60 | 32.81 | 4.62 [3.5-6.12] | 1.16 | -15.96 |
|  | 1002915 | Nephropathy toxic | 10/2,318 (0.43%) | 772/952,859 (0.08%) | 5.32 | 30.55 | 5.34 [3.88-7.35] | 1.12 | -14.82 |
|  | 1004355 | Thrombocytopenia | 43/2,318 (1.86%) | 7,877/952,859 (0.83%) | 2.24 | 28.50 | 2.27 [1.94-2.65] | 0.74 | -13.79 |
|  | 1001495 | Eosinophilia | 8/2,318 (0.35%) | 545/952,859 (0.06%) | 6.03 | 28.34 | 6.05 [4.24-8.65] | 1.04 | -13.72 |
|  | 1004004 | Sepsis | 37/2,318 (1.60%) | 6,442/952,859 (0.68%) | 2.36 | 27.71 | 2.38 [2.02-2.81] | 0.77 | -13.42 |
|  | 1003788 | Rash pustular | 3/2,318 (0.13%) | 82/952,859 (0.01%) | 15.04 | 25.57 | 15.06 [8.36-27.11] | 0.26 | -12.33 |
|  | 1007417 | Candida infection | 5/2,318 (0.22%) | 244/952,859 (0.03%) | 8.42 | 25.18 | 8.44 [5.37-13.27] | 0.76 | -12.15 |
|  | 1001370 | Drug hypersensitivity | 9/2,318 (0.39%) | 828/952,859 (0.09%) | 4.47 | 20.67 | 4.48 [3.2-6.27] | 0.87 | -9.86 |
|  | 1001868 | Granulocyte count decreased | 7/2,318 (0.30%) | 533/952,859 (0.06%) | 5.40 | 20.61 | 5.41 [3.7-7.92] | 0.83 | -9.85 |
|  | 1004721 | Venoocclusive liver disease | 8/2,318 (0.35%) | 782/952,859 (0.08%) | 4.21 | 16.31 | 4.22 [2.95-6.02] | 0.72 | -7.77 |
|  | 1000863 | Cholestasis | 7/2,318 (0.30%) | 636/952,859 (0.07%) | 4.52 | 15.69 | 4.54 [3.1-6.64] | 0.68 | -7.47 |
|  | 1003573 | Pneumonia staphylococcal | 4/2,318 (0.17%) | 228/952,859 (0.02%) | 7.21 | 15.36 | 7.22 [4.36-11.97] | 0.40 | -7.32 |
|  | 1001466 | Endocarditis | 5/2,318 (0.22%) | 360/952,859 (0.04%) | 5.71 | 14.79 | 5.72 [3.64-8.98] | 0.53 | -7.04 |
|  | 1001372 | Drug level increased | 7/2,318 (0.30%) | 672/952,859 (0.07%) | 4.28 | 14.33 | 4.29 [2.93-6.28] | 0.63 | -6.81 |
|  | 1004886 | Hypoacusis | 4/2,318 (0.17%) | 252/952,859 (0.03%) | 6.52 | 13.38 | 6.53 [3.95-10.82] | 0.35 | -6.36 |
|  | 1000682 | C-reactive protein increased | 15/2,318 (0.65%) | 2,424/952,859 (0.25%) | 2.54 | 12.50 | 2.55 [1.97-3.31] | 0.56 | -5.92 |
|  | 1002108 | Hypoproteinaemia | 3/2,318 (0.13%) | 161/952,859 (0.02%) | 7.66 | 11.13 | 7.67 [4.28-13.74] | 0.05 | -5.25 |
|  | 1005989 | Urine output decreased | 4/2,318 (0.17%) | 288/952,859 (0.03%) | 5.71 | 11.03 | 5.72 [3.45-9.46] | 0.27 | -5.24 |
|  | 1001868 | Granulocytopenia | 7/2,318 (0.30%) | 842/952,859 (0.09%) | 3.42 | 9.60 | 3.42 [2.34-5.01] | 0.42 | -4.57 |
|  | 1000519 | Blister | 5/2,318 (0.22%) | 500/952,859 (0.05%) | 4.11 | 8.78 | 4.12 [2.63-6.46] | 0.29 | -4.19 |
|  | 1005957 | Blood alkaline phosphatase increased | 8/2,318 (0.35%) | 1,095/952,859 (0.11%) | 3.00 | 8.72 | 3.01 [2.11-4.3] | 0.39 | -4.18 |
|  | 1005423 | Clostridium difficile infection | 4/2,318 (0.17%) | 337/952,859 (0.04%) | 4.88 | 8.65 | 4.89 [2.95-8.08] | 0.18 | -4.16 |
|  | 1003786 | Rash maculo-papular | 4/2,318 (0.17%) | 350/952,859 (0.04%) | 4.70 | 8.14 | 4.7 [2.84-7.78] | 0.15 | -3.91 |
|  | 1005101 | Staphylococcal bacteraemia | 3/2,318 (0.13%) | 201/952,859 (0.02%) | 6.14 | 8.14 | 6.14 [3.43-10.99] | -0.04 | -3.91 |
|  | 1003757 | Pustular psoriasis | 3/2,318 (0.13%) | 219/952,859 (0.02%) | 5.63 | 7.16 | 5.64 [3.15-10.08] | -0.08 | -3.42 |
|  | 1002741 | Metabolic acidosis | 5/2,318 (0.22%) | 591/952,859 (0.06%) | 3.48 | 6.47 | 3.48 [2.22-5.46] | 0.15 | -3.10 |
|  | 1004794 | White blood cell count increased | 8/2,318 (0.35%) | 1,265/952,859 (0.13%) | 2.60 | 6.32 | 2.61 [1.83-3.72] | 0.24 | -3.02 |
| Teicoplanin | 1003366 | Pancytopenia | 61/838 (7.28%) | 7,688/954,339 (0.81%) | 9.04 | 428.04 | 9.67 [8.46-11.05] | 2.66 | -212.51 |
|  | 1008673 | Vancomycin infusion reaction | 3/838 (0.36%) | 19/954,339 (<0.01%) | 179.82 | 319.12 | 180.46 [96.86-336.2] | 0.45 | -158.31 |
|  | 1003552 | Platelet count decreased | 98/838 (11.69%) | 22,320/954,339 (2.34%) | 5.00 | 315.69 | 5.53 [4.97-6.16] | 2.00 | -156.88 |
|  | 1004355 | Thrombocytopenia | 45/838 (5.37%) | 7,875/954,339 (0.83%) | 6.51 | 204.82 | 6.82 [5.85-7.95] | 2.15 | -101.63 |
|  | 1005413 | Stenotrophomonas infection | 4/838 (0.48%) | 70/954,339 (0.01%) | 65.08 | 181.93 | 65.38 [39.06-109.46] | 0.94 | -90.28 |
|  | 1001344 | Disseminated intravascular coagulation | 30/838 (3.58%) | 4,584/954,339 (0.48%) | 7.45 | 160.95 | 7.69 [6.38-9.27] | 2.16 | -79.87 |
|  | 1001868 | Granulocytopenia | 12/838 (1.43%) | 837/954,339 (0.09%) | 16.33 | 155.57 | 16.55 [12.35-22.18] | 2.16 | -77.28 |
|  | 1004794 | White blood cell count decreased | 56/838 (6.68%) | 14,425/954,339 (1.51%) | 4.42 | 146.51 | 4.67 [4.06-5.36] | 1.72 | -72.82 |
|  | 1003766 | Pyrexia | 72/838 (8.59%) | 21,792/954,339 (2.28%) | 3.76 | 146.17 | 4.02 [3.56-4.55] | 1.55 | -72.75 |
|  | 1004422 | Toxic epidermal necrolysis | 22/838 (2.63%) | 3,867/954,339 (0.41%) | 6.48 | 96.37 | 6.63 [5.34-8.23] | 1.84 | -47.73 |
|  | 1003784 | Rash | 46/838 (5.49%) | 13,562/954,339 (1.42%) | 3.86 | 95.79 | 4.03 [3.46-4.69] | 1.49 | -47.52 |
|  | 1004256 | Superinfection | 3/838 (0.36%) | 70/954,339 (0.01%) | 48.81 | 92.74 | 48.98 [27.13-88.41] | 0.44 | -46.05 |
|  | 1006933 | Acute kidney injury | 39/838 (4.65%) | 10,662/954,339 (1.12%) | 4.17 | 91.37 | 4.32 [3.67-5.09] | 1.54 | -45.43 |
|  | 1000150 | Agranulocytosis | 23/838 (2.74%) | 4,561/954,339 (0.48%) | 5.74 | 85.39 | 5.88 [4.75-7.26] | 1.73 | -42.47 |
|  | 1001967 | Hepatic function abnormal | 58/838 (6.92%) | 21,481/954,339 (2.25%) | 3.07 | 80.75 | 3.23 [2.82-3.7] | 1.23 | -40.18 |
|  | 1001494 | Eosinophil count increased | 9/838 (1.07%) | 898/954,339 (0.09%) | 11.41 | 74.73 | 11.53 [8.23-16.14] | 1.63 | -37.19 |
|  | 1006223 | Renal impairment | 47/838 (5.61%) | 16,679/954,339 (1.75%) | 3.21 | 70.32 | 3.34 [2.87-3.88] | 1.24 | -35.05 |
|  | 1007158 | Haemophagocytic lymphohistiocytosis | 11/838 (1.31%) | 1,556/954,339 (0.16%) | 8.05 | 60.72 | 8.14 [6.01-11.04] | 1.57 | -30.24 |
|  | 1005957 | Blood alkaline phosphatase increased | 9/838 (1.07%) | 1,094/954,339 (0.11%) | 9.37 | 58.75 | 9.46 [6.76-13.24] | 1.49 | -29.28 |
|  | 1003843 | Renal failure | 16/838 (1.91%) | 3,199/954,339 (0.34%) | 5.70 | 57.24 | 5.79 [4.49-7.45] | 1.52 | -28.56 |
|  | 1001868 | Granulocyte count decreased | 6/838 (0.72%) | 534/954,339 (0.06%) | 12.80 | 53.40 | 12.88 [8.53-19.45] | 1.20 | -26.73 |
|  | 1002084 | Hyperthermia | 6/838 (0.72%) | 555/954,339 (0.06%) | 12.31 | 51.03 | 12.39 [8.21-18.71] | 1.19 | -25.56 |
|  | 1001370 | Drug hypersensitivity | 7/838 (0.84%) | 830/954,339 (0.09%) | 9.60 | 45.35 | 9.68 [6.61-14.17] | 1.24 | -22.70 |
|  | 1002467 | Liver disorder | 31/838 (3.7%) | 12,518/954,339 (1.31%) | 2.82 | 34.99 | 2.89 [2.41-3.47] | 0.96 | -17.47 |
|  | 1001368 | Drug eruption | 25/838 (2.98%) | 9,960/954,339 (1.04%) | 2.86 | 28.60 | 2.92 [2.38-3.57] | 0.91 | -14.21 |
|  | 1003364 | Pancreatitis acute | 11/838 (1.31%) | 2,718/954,339 (0.28%) | 4.61 | 27.55 | 4.66 [3.44-6.31] | 1.05 | -13.70 |
|  | 1000563 | Blood lactate dehydrogenase increased | 6/838 (0.72%) | 1,060/954,339 (0.11%) | 6.45 | 22.32 | 6.49 [4.3-9.78] | 0.81 | -11.05 |
|  | 1001245 | Dermatitis exfoliative generalised | 5/838 (0.60%) | 767/954,339 (0.08%) | 7.42 | 21.61 | 7.46 [4.76-11.7] | 0.70 | -10.71 |
|  | 1001495 | Eosinophilia | 4/838 (0.48%) | 549/954,339 (0.06%) | 8.30 | 18.76 | 8.33 [5.04-13.78] | 0.48 | -9.27 |
|  | 1001515 | Erythema | 16/838 (1.91%) | 6,218/954,339 (0.65%) | 2.93 | 18.53 | 2.97 [2.31-3.82] | 0.76 | -9.18 |
|  | 1004203 | Stevens-Johnson syndrome | 17/838 (2.03%) | 6,856/954,339 (0.72%) | 2.82 | 18.33 | 2.86 [2.24-3.66] | 0.74 | -9.10 |
|  | 1000536 | Blood bilirubin increased | 7/838 (0.84%) | 1,661/954,339 (0.17%) | 4.80 | 17.38 | 4.83 [3.3-7.07] | 0.74 | -8.62 |
|  | 1000585 | Blood urea increased | 5/838 (0.60%) | 1,015/954,339 (0.11%) | 5.61 | 14.55 | 5.64 [3.6-8.84] | 0.52 | -7.20 |
|  | 1002057 | Hyperbilirubinaemia | 4/838 (0.48%) | 759/954,339 (0.08%) | 6.00 | 11.99 | 6.03 [3.65-9.96] | 0.31 | -5.90 |
|  | 1007736 | Multiple organ dysfunction syndrome | 7/838 (0.84%) | 2,098/954,339 (0.22%) | 3.80 | 11.76 | 3.82 [2.61-5.59] | 0.53 | -5.80 |
|  | 1003712 | Pseudomembranous colitis | 5/838 (0.60%) | 1,169/954,339 (0.12%) | 4.87 | 11.72 | 4.89 [3.12-7.67] | 0.42 | -5.80 |
|  | 1001769 | Gamma-glutamyltransferase increased | 5/838 (0.60%) | 1,354/954,339 (0.14%) | 4.21 | 9.20 | 4.22 [2.7-6.62] | 0.31 | -4.45 |
|  | 1004879 | Acute generalised exanthematous pustulosis | 4/838 (0.48%) | 965/954,339 (0.1%) | 4.72 | 8.28 | 4.74 [2.87-7.83] | 0.16 | -4.06 |
|  | 1000105 | Acute respiratory distress syndrome | 5/838 (0.60%) | 1,466/954,339 (0.15%) | 3.88 | 8.00 | 3.9 [2.49-6.11] | 0.25 | -3.93 |
|  | 1003842 | Renal disorder | 8/838 (0.95%) | 3,261/954,339 (0.34%) | 2.79 | 7.51 | 2.81 [1.97-4.01] | 0.32 | -3.70 |
|  | 1000572 | Blood potassium decreased | 3/838 (0.36%) | 598/954,339 (0.06%) | 5.71 | 7.39 | 5.73 [3.21-10.23] | -0.07 | -3.65 |
| Arbekacin | 1000585 | Blood urea increased | 11/227 (4.85%) | 1,009/954,950 (0.11%) | 45.86 | 434.63 | 48.15 [35.29-65.69] | 2.49 | -215.76 |
|  | 1006223 | Renal impairment | 35/227 (15.42%) | 16,691/954,950 (1.75%) | 8.82 | 238.65 | 10.25 [8.53-12.32] | 2.40 | -118.16 |
|  | 1006933 | Acute kidney injury | 27/227 (11.89%) | 10,674/954,950 (1.12%) | 10.64 | 228.29 | 11.94 [9.73-14.66] | 2.48 | -113.37 |
|  | 1003842 | Renal disorder | 14/227 (6.17%) | 3,255/954,950 (0.34%) | 18.09 | 209.13 | 19.22 [14.58-25.34] | 2.39 | -104.26 |
|  | 1003843 | Renal failure | 12/227 (5.29%) | 3,203/954,950 (0.34%) | 15.76 | 151.40 | 16.58 [12.32-22.32] | 2.14 | -75.41 |
|  | 1000548 | Blood creatinine increased | 7/227 (3.08%) | 2,484/954,950 (0.26%) | 11.85 | 59.13 | 12.2 [8.31-17.92] | 1.36 | -29.11 |
|  | 1004422 | Toxic epidermal necrolysis | 8/227 (3.52%) | 3,881/954,950 (0.41%) | 8.67 | 46.99 | 8.95 [6.24-12.84] | 1.31 | -23.15 |
|  | 1002469 | Liver function test abnormal | 3/227 (1.32%) | 642/954,950 (0.07%) | 19.66 | 35.96 | 19.91 [11.12-35.65] | 0.33 | -17.60 |
|  | 1001967 | Hepatic function abnormal | 18/227 (7.93%) | 21,521/954,950 (2.25%) | 3.52 | 30.65 | 3.74 [2.92-4.78] | 1.02 | -15.03 |
|  | 1001344 | Disseminated intravascular coagulation | 7/227 (3.08%) | 4,607/954,950 (0.48%) | 6.39 | 26.76 | 6.56 [4.47-9.64] | 0.96 | -13.10 |
|  | 1003552 | Platelet count decreased | 16/227 (7.05%) | 22,402/954,950 (2.35%) | 3.00 | 19.89 | 3.16 [2.44-4.09] | 0.78 | -9.73 |
|  | 1005957 | Blood alkaline phosphatase increased | 3/227 (1.32%) | 1,100/954,950 (0.12%) | 11.47 | 19.13 | 11.61 [6.49-20.78] | 0.20 | -9.38 |
|  | 1003712 | Pseudomembranous colitis | 3/227 (1.32%) | 1,171/954,950 (0.12%) | 10.78 | 17.71 | 10.91 [6.1-19.52] | 0.18 | -8.69 |
|  | 1001769 | Gamma-glutamyltransferase increased | 3/227 (1.32%) | 1,356/954,950 (0.14%) | 9.31 | 14.70 | 9.42 [5.26-16.85] | 0.13 | -7.20 |
|  | 1000203 | Anaemia | 10/227 (4.41%) | 15,085/954,950 (1.58%) | 2.79 | 9.90 | 2.87 [2.08-3.97] | 0.44 | -4.85 |
|  | 1004355 | Thrombocytopenia | 6/227 (2.64%) | 7,914/954,950 (0.83%) | 3.19 | 7.01 | 3.25 [2.15-4.91] | 0.23 | -3.55 |
|  | 1000682 | C-reactive protein increased | 3/227 (1.32%) | 2,436/954,950 (0.26%) | 5.18 | 6.38 | 5.24 [2.93-9.37] | -0.12 | -3.23 |
| Daptomycin | 1001496 | Eosinophilic pneumonia | 66/552 (11.96%) | 1,075/954,625 (0.11%) | 106.18 | 6387.39 | 120.46 [105.28-137.83] | 5.01 | -3192.72 |
|  | 1006816 | Cardiac valve rupture | 3/552 (0.54%) | 5/954,625 (<0.01%) | 1037.64 | 1347.66 | 1043.3 [502-2168.28] | 0.38 | -672.77 |
|  | 1000547 | Blood creatine phosphokinase increased | 51/552 (9.24%) | 3,904/954,625 (0.41%) | 22.59 | 1021.89 | 24.79 [21.38-28.74] | 3.62 | -510.15 |
|  | 1005283 | Eosinophilic pneumonia acute | 7/552 (1.27%) | 156/954,625 (0.02%) | 77.60 | 435.94 | 78.58 [53.27-115.92] | 1.89 | -217.04 |
|  | 1001836 | Glomerulonephritis acute | 3/552 (0.54%) | 32/954,625 (<0.01%) | 162.13 | 304.21 | 163.01 [88.99-298.61] | 0.47 | -151.22 |
|  | 1001466 | Endocarditis | 7/552 (1.27%) | 358/954,625 (0.04%) | 33.81 | 187.69 | 34.24 [23.32-50.27] | 1.75 | -93.06 |
|  | 1003902 | Rhabdomyolysis | 31/552 (5.62%) | 7,991/954,625 (0.84%) | 6.71 | 145.60 | 7.05 [5.86-8.48] | 2.05 | -72.02 |
|  | 1006223 | Renal impairment | 42/552 (7.61%) | 16,684/954,625 (1.75%) | 4.35 | 106.77 | 4.63 [3.94-5.44] | 1.62 | -52.68 |
|  | 1005986 | Drug resistance | 5/552 (0.91%) | 361/954,625 (0.04%) | 23.95 | 87.03 | 24.16 [15.37-37.98] | 1.16 | -42.80 |
|  | 1005797 | Toxic skin eruption | 10/552 (1.81%) | 1,911/954,625 (0.20%) | 9.05 | 63.57 | 9.2 [6.68-12.67] | 1.57 | -31.08 |
|  | 1005101 | Staphylococcal bacteraemia | 3/552 (0.54%) | 201/954,625 (0.02%) | 25.81 | 48.17 | 25.95 [14.48-46.49] | 0.38 | -23.52 |
|  | 1005643 | Staphylococcal sepsis | 4/552 (0.72%) | 422/954,625 (0.04%) | 16.39 | 43.05 | 16.5 [9.97-27.33] | 0.73 | -21.01 |
|  | 1001370 | Drug ineffective | 10/552 (1.81%) | 2,611/954,625 (0.27%) | 6.62 | 42.24 | 6.73 [4.89-9.26] | 1.32 | -20.65 |
|  | 1000585 | Blood urea increased | 6/552 (1.09%) | 1,014/954,625 (0.11%) | 10.23 | 40.97 | 10.33 [6.85-15.6] | 1.10 | -20.05 |
|  | 1004422 | Toxic epidermal necrolysis | 12/552 (2.17%) | 3,877/954,625 (0.41%) | 5.35 | 38.27 | 5.45 [4.07-7.3] | 1.26 | -18.71 |
|  | 1007051 | Hypoxic-ischaemic encephalopathy | 4/552 (0.72%) | 553/954,625 (0.06%) | 12.51 | 31.41 | 12.59 [7.61-20.84] | 0.65 | -15.28 |
|  | 1003566 | Pneumonia | 29/552 (5.25%) | 19,064/954,625 (2.00%) | 2.63 | 28.23 | 2.72 [2.25-3.29] | 0.84 | -13.72 |
|  | 1003329 | Overdose | 5/552 (0.91%) | 983/954,625 (0.10%) | 8.80 | 27.08 | 8.87 [5.65-13.91] | 0.79 | -13.16 |
|  | 1007736 | Multiple organ dysfunction syndrome | 7/552 (1.27%) | 2,098/954,625 (0.22%) | 5.77 | 23.01 | 5.83 [3.98-8.54] | 0.89 | -11.25 |
|  | 1002098 | Hypogammaglobulinaemia | 3/552 (0.54%) | 389/954,625 (0.04%) | 13.34 | 22.84 | 13.4 [7.5-23.97] | 0.25 | -11.19 |
|  | 1003784 | Rash | 20/552 (3.62%) | 13,588/954,625 (1.42%) | 2.55 | 17.48 | 2.6 [2.07-3.27] | 0.67 | -8.55 |
|  | 1001494 | Eosinophil count increased | 4/552 (0.72%) | 903/954,625 (0.09%) | 7.66 | 16.92 | 7.71 [4.66-12.75] | 0.44 | -8.29 |
|  | 1000755 | Cardiac failure acute | 5/552 (0.91%) | 1,476/954,625 (0.15%) | 5.86 | 15.55 | 5.9 [3.76-9.26] | 0.55 | -7.65 |
|  | 1004203 | Stevens-Johnson syndrome | 12/552 (2.17%) | 6,861/954,625 (0.72%) | 3.02 | 14.38 | 3.07 [2.29-4.11] | 0.65 | -7.10 |
|  | 1003866 | Respiratory arrest | 4/552 (0.72%) | 1,030/954,625 (0.11%) | 6.72 | 14.12 | 6.76 [4.09-11.17] | 0.37 | -6.98 |
|  | 1005957 | Blood alkaline phosphatase increased | 4/552 (0.72%) | 1,099/954,625 (0.12%) | 6.29 | 12.88 | 6.33 [3.83-10.47] | 0.34 | -6.34 |
|  | 1001966 | Hepatic failure | 5/552 (0.91%) | 1,760/954,625 (0.18%) | 4.91 | 11.90 | 4.95 [3.16-7.76] | 0.43 | -5.87 |
|  | 1005808 | Staphylococcal infection | 3/552 (0.54%) | 670/954,625 (0.07%) | 7.74 | 11.47 | 7.78 [4.36-13.9] | 0.07 | -5.68 |
|  | 1003842 | Renal disorder | 7/552 (1.27%) | 3,262/954,625 (0.34%) | 3.71 | 11.30 | 3.75 [2.56-5.48] | 0.50 | -5.60 |
|  | 1004794 | White blood cell count increased | 4/552 (0.72%) | 1,269/954,625 (0.13%) | 5.45 | 10.41 | 5.48 [3.32-9.06] | 0.25 | -5.19 |
|  | 1004364 | Thrombotic microangiopathy | 4/552 (0.72%) | 1,381/954,625 (0.14%) | 5.01 | 9.12 | 5.04 [3.05-8.33] | 0.20 | -4.56 |
|  | 1004007 | Septic shock | 6/552 (1.09%) | 2,859/954,625 (0.30%) | 3.63 | 8.96 | 3.66 [2.43-5.52] | 0.36 | -4.48 |
|  | 1004004 | Sepsis | 10/552 (1.81%) | 6,469/954,625 (0.68%) | 2.67 | 8.91 | 2.7 [1.96-3.72] | 0.41 | -4.47 |
|  | 1004789 | Weight increased | 3/552 (0.54%) | 806/954,625 (0.08%) | 6.44 | 8.85 | 6.47 [3.62-11.55] | -0.01 | -4.45 |
|  | 1006747 | Organising pneumonia | 4/552 (0.72%) | 1,424/954,625 (0.15%) | 4.86 | 8.69 | 4.89 [2.96-8.08] | 0.18 | -4.38 |
|  | 1001026 | Condition aggravated | 6/552 (1.09%) | 3,282/954,625 (0.34%) | 3.16 | 6.85 | 3.19 [2.11-4.8] | 0.23 | -3.46 |
|  | 1004879 | Acute generalised exanthematous pustulosis | 3/552 (0.54%) | 966/954,625 (0.10%) | 5.37 | 6.73 | 5.39 [3.02-9.63] | -0.10 | -3.41 |
|  | 1001515 | Erythema | 9/552 (1.63%) | 6,225/954,625 (0.65%) | 2.50 | 6.70 | 2.53 [1.8-3.53] | 0.27 | -3.40 |
| Linezolid | 1003552 | Platelet count decreased | 532/1,750 (30.4%) | 21,886/953,427 (2.30%) | 13.24 | 6007.72 | 18.59 [17.64-19.59] | 3.54 | -3002.43 |
|  | 1003366 | Pancytopenia | 228/1,750 (13.03%) | 7,521/953,427 (0.79%) | 16.52 | 3236.89 | 18.84 [17.53-20.25] | 3.74 | -1617.40 |
|  | 1004355 | Thrombocytopenia | 172/1,750 (9.83%) | 7,748/953,427 (0.81%) | 12.09 | 1715.83 | 13.3 [12.27-14.43] | 3.28 | -856.96 |
|  | 1006132 | Optic nerve disorder | 14/1,750 (0.80%) | 53/953,427 (0.01%) | 143.91 | 1460.60 | 145.07 [107.31-196.1] | 3.01 | -729.55 |
|  | 1002103 | Hyponatraemia | 89/1,750 (5.09%) | 4,173/953,427 (0.44%) | 11.62 | 839.12 | 12.19 [10.92-13.6] | 3.08 | -418.76 |
|  | 1000203 | Anaemia | 156/1,750 (8.91%) | 14,939/953,427 (1.57%) | 5.69 | 601.57 | 6.15 [5.65-6.69] | 2.25 | -300.00 |
|  | 1002858 | Myelosuppression | 99/1,750 (5.66%) | 7,633/953,427 (0.80%) | 7.07 | 507.09 | 7.43 [6.7-8.25] | 2.47 | -252.82 |
|  | 1003815 | Red blood cell count decreased | 29/1,750 (1.66%) | 763/953,427 (0.08%) | 20.71 | 505.57 | 21.04 [17.39-25.46] | 3.14 | -252.19 |
|  | 1008045 | Product prescribing issue | 4/1,750 (0.23%) | 11/953,427 (0%) | 198.11 | 439.59 | 198.57 [110.69-356.2] | 0.91 | -219.25 |
|  | 1006132 | Optic neuropathy | 9/1,750 (0.51%) | 119/953,427 (0.01%) | 41.20 | 291.90 | 41.41 [29.28-58.56] | 2.15 | -145.40 |
|  | 1002367 | Lactic acidosis | 35/1,750 (2.00%) | 1,946/953,427 (0.20%) | 9.80 | 263.60 | 9.98 [8.4-11.85] | 2.53 | -131.29 |
|  | 1006627 | Cytopenia | 28/1,750 (1.60%) | 1,359/953,427 (0.14%) | 11.23 | 245.95 | 11.39 [9.4-13.81] | 2.56 | -122.51 |
|  | 1001888 | Haemoglobin decreased | 50/1,750 (2.86%) | 5,249/953,427 (0.55%) | 5.19 | 164.30 | 5.31 [4.6-6.14] | 1.90 | -81.63 |
|  | 1007647 | Product use in unapproved indication | 12/1,750 (0.69%) | 415/953,427 (0.04%) | 15.75 | 147.17 | 15.86 [11.82-21.27] | 2.13 | -73.08 |
|  | 1005376 | Off label use | 24/1,750 (1.37%) | 1,662/953,427 (0.17%) | 7.87 | 135.36 | 7.96 [6.47-9.79] | 2.10 | -67.19 |
|  | 1007630 | Product use issue | 9/1,750 (0.51%) | 289/953,427 (0.03%) | 16.97 | 116.13 | 17.05 [12.14-23.94] | 1.84 | -57.56 |
|  | 1003094 | Optic neuritis | 9/1,750 (0.51%) | 337/953,427 (0.04%) | 14.55 | 97.82 | 14.62 [10.42-20.51] | 1.76 | -48.37 |
|  | 1004794 | White blood cell count decreased | 69/1,750 (3.94%) | 14,412/953,427 (1.51%) | 2.61 | 67.54 | 2.67 [2.36-3.02] | 1.04 | -33.10 |
|  | 1002933 | Neuropathy peripheral | 35/1,750 (2.00%) | 5,232/953,427 (0.55%) | 3.64 | 64.47 | 3.7 [3.12-4.39] | 1.33 | -31.73 |
|  | 1002066 | Hyperlactacidaemia | 4/1,750 (0.23%) | 96/953,427 (0.01%) | 22.70 | 60.16 | 22.75 [13.65-37.92] | 0.79 | -29.58 |
|  | 1003712 | Pseudomembranous colitis | 13/1,750 (0.74%) | 1,161/953,427 (0.12%) | 6.10 | 49.95 | 6.14 [4.64-8.12] | 1.45 | -24.53 |
|  | 1004256 | Superinfection | 3/1,750 (0.17%) | 70/953,427 (0.01%) | 23.35 | 41.94 | 23.39 [12.96-42.19] | 0.34 | -20.58 |
|  | 1002852 | Myelitis | 5/1,750 (0.29%) | 237/953,427 (0.02%) | 11.49 | 37.19 | 11.52 [7.33-18.12] | 0.91 | -18.17 |
|  | 1004886 | Hypoacusis | 5/1,750 (0.29%) | 251/953,427 (0.03%) | 10.85 | 34.72 | 10.88 [6.92-17.1] | 0.88 | -16.93 |
|  | 1001883 | Haematocrit decreased | 5/1,750 (0.29%) | 280/953,427 (0.03%) | 9.73 | 30.37 | 9.75 [6.21-15.33] | 0.83 | -14.78 |
|  | 1004757 | Visual impairment | 9/1,750 (0.51%) | 867/953,427 (0.09%) | 5.66 | 29.70 | 5.68 [4.06-7.95] | 1.09 | -14.48 |
|  | 1002741 | Metabolic acidosis | 7/1,750 (0.40%) | 589/953,427 (0.06%) | 6.47 | 26.85 | 6.5 [4.44-9.51] | 0.97 | -13.06 |
|  | 1004159 | Spinal osteoarthritis | 3/1,750 (0.17%) | 109/953,427 (0.01%) | 14.99 | 25.71 | 15.02 [8.36-26.98] | 0.26 | -12.49 |
|  | 1001794 | Gastrointestinal disorder | 6/1,750 (0.34%) | 457/953,427 (0.05%) | 7.15 | 25.57 | 7.17 [4.75-10.83] | 0.88 | -12.44 |
|  | 1001886 | Haematuria | 11/1,750 (0.63%) | 1,403/953,427 (0.15%) | 4.27 | 24.23 | 4.29 [3.17-5.82] | 0.97 | -11.77 |
|  | 1000296 | Aplasia pure red cell | 7/1,750 (0.40%) | 668/953,427 (0.07%) | 5.71 | 22.46 | 5.73 [3.91-8.38] | 0.87 | -10.87 |
|  | 1000580 | Blood sodium decreased | 5/1,750 (0.29%) | 392/953,427 (0.04%) | 6.95 | 19.61 | 6.97 [4.44-10.93] | 0.65 | -9.45 |
|  | 1001438 | Electrocardiogram QT prolonged | 18/1,750 (1.03%) | 3,499/953,427 (0.37%) | 2.80 | 19.08 | 2.82 [2.22-3.58] | 0.76 | -9.21 |
|  | 1006093 | Adverse event | 4/1,750 (0.23%) | 293/953,427 (0.03%) | 7.44 | 16.09 | 7.45 [4.5-12.34] | 0.42 | -7.73 |
|  | 1000586 | Blood uric acid increased | 4/1,750 (0.23%) | 298/953,427 (0.03%) | 7.31 | 15.73 | 7.33 [4.43-12.13] | 0.41 | -7.59 |
|  | 1004388 | Tinnitus | 4/1,750 (0.23%) | 344/953,427 (0.04%) | 6.34 | 12.88 | 6.35 [3.84-10.5] | 0.34 | -6.22 |
|  | 1001741 | Full blood count decreased | 3/1,750 (0.17%) | 197/953,427 (0.02%) | 8.30 | 12.45 | 8.31 [4.64-14.87] | 0.09 | -6.00 |
|  | 1002057 | Hyperbilirubinaemia | 6/1,750 (0.34%) | 757/953,427 (0.08%) | 4.32 | 12.07 | 4.33 [2.87-6.53] | 0.50 | -5.82 |
|  | 1005160 | Platelet count increased | 3/1,750 (0.17%) | 202/953,427 (0.02%) | 8.09 | 12.04 | 8.1 [4.53-14.5] | 0.08 | -5.81 |
|  | 1001466 | Endocarditis | 4/1,750 (0.23%) | 361/953,427 (0.04%) | 6.04 | 12.01 | 6.05 [3.66-10.01] | 0.31 | -5.81 |
|  | 1005986 | Drug resistance | 4/1,750 (0.23%) | 362/953,427 (0.04%) | 6.02 | 11.97 | 6.03 [3.65-9.98] | 0.31 | -5.80 |
|  | 1004010 | Serotonin syndrome | 7/1,750 (0.40%) | 1,003/953,427 (0.11%) | 3.80 | 11.72 | 3.81 [2.61-5.58] | 0.53 | -5.70 |
|  | 1008249 | Taste disorder | 5/1,750 (0.29%) | 560/953,427 (0.06%) | 4.86 | 11.63 | 4.88 [3.11-7.65] | 0.42 | -5.66 |
|  | 1006471 | Portal venous gas | 3/1,750 (0.17%) | 209/953,427 (0.02%) | 7.82 | 11.50 | 7.83 [4.38-14.02] | 0.06 | -5.61 |
|  | 1006147 | Pseudomonas infection | 3/1,750 (0.17%) | 240/953,427 (0.03%) | 6.81 | 9.50 | 6.82 [3.81-12.2] | 0.01 | -4.64 |
|  | 1002469 | Liver function test abnormal | 5/1,750 (0.29%) | 640/953,427 (0.07%) | 4.26 | 9.34 | 4.27 [2.72-6.69] | 0.32 | -4.56 |
|  | 1000105 | Acute respiratory distress syndrome | 8/1,750 (0.46%) | 1,463/953,427 (0.15%) | 2.98 | 8.60 | 2.99 [2.09-4.26] | 0.38 | -4.21 |
|  | 1000048 | Acidosis | 3/1,750 (0.17%) | 278/953,427 (0.03%) | 5.88 | 7.67 | 5.89 [3.29-10.53] | -0.06 | -3.74 |
|  | 1001370 | Drug ineffective | 11/1,750 (0.63%) | 2,610/953,427 (0.27%) | 2.30 | 6.79 | 2.3 [1.7-3.12] | 0.28 | -3.28 |
|  | 1000572 | Blood potassium increased | 5/1,750 (0.29%) | 783/953,427 (0.08%) | 3.48 | 6.49 | 3.49 [2.22-5.46] | 0.16 | -3.12 |
| Tedizolid | 1004355 | Thrombocytopenia | 8/39 (20.51%) | 7,912/955,138 (0.83%) | 24.76 | 160.61 | 30.9 [20.78-45.94] | 1.80 | -80.27 |
|  | 1002858 | Myelosuppression | 3/39 (7.69%) | 7,729/955,138 (0.81%) | 9.51 | 15.24 | 10.21 [5.6-18.63] | 0.11 | -8.05 |
|  | 1003366 | Pancytopenia | 3/39 (7.69%) | 7,746/955,138 (0.81%) | 9.49 | 15.19 | 10.19 [5.59-18.59] | 0.11 | -8.05 |
|  | 1003552 | Platelet count decreased | 5/39 (12.82%) | 22,413/955,138 (2.35%) | 5.46 | 14.38 | 6.12 [3.79-9.88] | 0.45 | -7.71 |

AE, adverse event; PRR, proportional reporting ratio; SMQ, Standardized MedDRA^®^ Query.
